# Tuberculosis outbreak in a German daycare center

**DOI:** 10.1007/s15010-025-02655-0

**Published:** 2025-10-04

**Authors:** Cornelia Feiterna-Sperling, Julia von Hake, Birgit Lala, Mirjam Völler, Lujin Zaidan-Braun, Luise Martin, Annette Günther, Dinah von Schöning, Renate Krüger

**Affiliations:** 1 Department of Pediatric Respiratory Medicine, Immunology, and Intensive Care Medicine, Charité - Universitätsmedizin Berlin, corporate member of Freie Universität Berlin and Humboldt-Universität zu Berlin, Berlin, Germany; 2 Health Department, Bezirksamt Lichtenberg von Berlin, Berlin, Germany; 3 Department of Pediatric Radiology, Charité-Universitätsmedizin Berlin, Berlin, Germany; 4 Medical Directorate, Charité - Universitätsmedizin Berlin, corporate member of Freie Universität Berlin and Humboldt-Universität zu Berlin, Berlin, Germany; 5 Clinic for Pediatrics and Adolescent Medicine, Helios Klinikum Emil von Behring, Berlin, Germany; 6 Department of Microbiology and Hygiene, Labor Berlin - Charité Vivantes GmbH, Berlin, Germany; 7 Institute for Medical Microbiology and Virology, University Hospital Leipzig, Leipzig, Germany; 8 Augustenburger Platz 1, 13353 Berlin, Germany

**Keywords:** Tuberculosis outbreak, Index case, Contact investigation, Daycare center, Tuberculin skin test, Interferon-gamma release assay

## Abstract

**Purpose:**

Young children who are exposed to people with infectious tuberculosis (TB) have an increased risk of developing TB disease following infection. The risk of infection and disease progression can be minimized by prompt identification of TB-exposed individuals and initiation of prophylactic or preventive treatment.

**Methods:**

We report on a TB outbreak in a daycare center in Berlin, Germany following a delayed diagnosis of cavitary pulmonary TB in a childhood educator. We describe contact investigation, diagnostic, prophylactic, preventive and therapeutic measures in 62 TB-exposed children (median age 3.9 years), including 30 with prolonged TB exposure.

**Results:**

The initial examination took place 5–16 days after the index patient was diagnosed with TB. Ten of the 30 children with intensive contact became infected, six (median age 2.7 years) developed pulmonary TB. Three of these children had a concurrent influenza infection, which may have contributed to disease progression. No child without prolonged exposure to the index patient developed disease.

**Conclusion:**

Early diagnosis of TB in adult patients, especially those with persistent cough, is crucial to prevent TB in vulnerable infants. Close collaboration between public health departments and specialized facilities is essential for the effective control of TB outbreaks.

## Introduction

In 2022, an estimated 10.6 million people worldwide contracted tuberculosis (TB), including approximately 1.3 million children [1]. Although most TB cases occur in low- and middle-income countries, TB also poses a public health challenge in low-incidence countries, especially when vulnerable populations are affected. In Germany, 4076 TB cases were registered in 2022 (incidence 4.9 cases per 100,000 inhabitants). However, the incidence among non-German nationals (25.1 cases/100,000) was 16 times higher than among individuals with German nationality (incidence 1.5/100,000). Children and adolescents < 15 years of age accounted for a small proportion of all cases with 190 TB cases (1.6/100,000 children) and the highest incidence (86 TB cases) in young children < 5 years of age (2.2/100,000) [2]. In general, TB exposed children < 5 years of age have the highest risk of progression to TB disease following infection with *Mycobacterium tuberculosis* (*M.tb*). Progression is mostly more rapid than in adults and associated with severe forms such as TB meningitis and miliary TB [3, 4]. Therefore, children in this age group should be promptly examined after TB exposure and receive prophylactic or preventive treatment to effectively reduce the risk of infection and/or disease [5–7]. However, there are repeated cases of infection with *M.tb* and TB disease in children in daycare centers as a result of prolonged TB exposure by infectious staff members [8–11]. This article describes a TB outbreak in a Berlin daycare center resulting from a delayed TB diagnosis in a symptomatic employee. The report also describes the challenges of contact investigations and the clinical management of pediatric TB contacts.

## Materials and methods

### Index case

In December 2022, a 27 year-old German childhood educator was diagnosed with fully sensitive cavitary pulmonary TB, who had been symptomatic for more than six months and had undergone multiple medical examinations. The only TB risk factors identified for the educator were an Interrail tour at the age of 19 and a vacation (less than 4 weeks) in Tunisia as a child. In Germany, testing for TB infection or disease is not required prior to employment or attendance of daycare centers or schools, given that no risk factors were reported. Therefore, no pre-employment testing of the childhood educator had been performed.

Following TB diagnosis, the educator was treated for 10 months and the TB healed with scarring residuals. Contact investigation was carried out in accordance to current recommendations [12].

### Study population

**Fig. 1:**
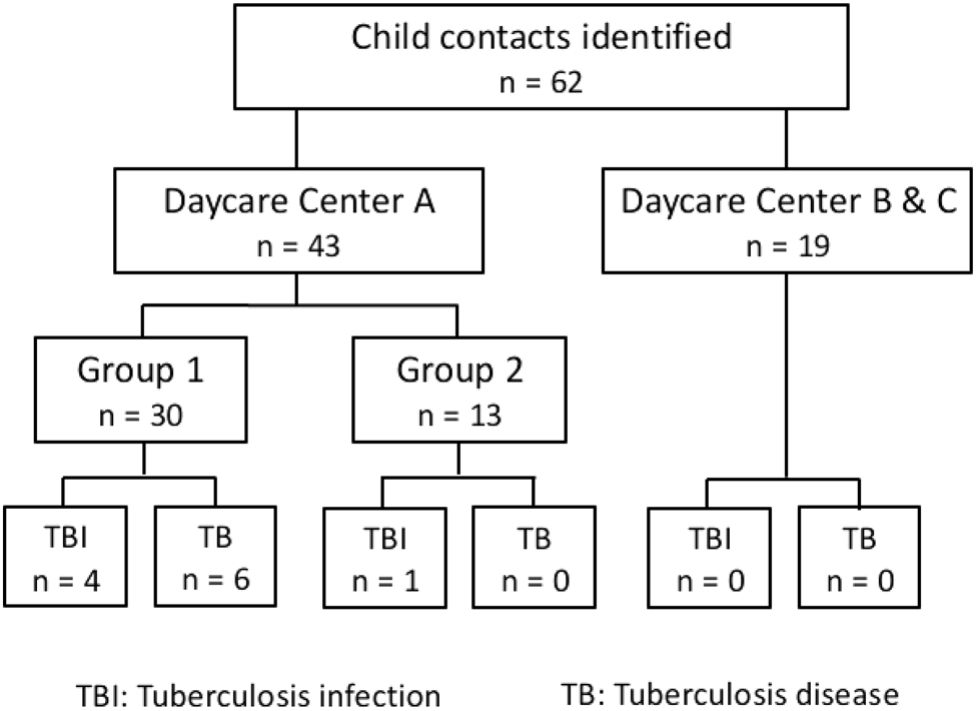
Flow diagram, from identification to diagnosis

All children in the daycare center (daycare center A, see flowchart, Fig. 1) with contact to the index patient at the time of diagnosis or in the previous six months were examined. The children were divided into groups according to the date of the last contact and the intensity of contact measured in time the educator spent with the child. Priority was given to the group of the daycare center, referred to as “daycare center A group 1”, in which the index patient had worked up to the TB diagnosis. This was followed by examinations of the group from the same daycare center where the last contact had been six months before the index patient was diagnosed with TB (“daycare center A group 2”) and of children from two other daycare centers (daycare center B and daycare center C), each of which had only had a brief contact (maximum 2 h, 10 days before TB diagnosis) as part of an internship of the index (Fig. 1).

### Initial examination

Children in all three groups were primarily examined by employees of the public health department; a medical history was taken and an immune-based TB test was carried out in each case.

### Diagnostic tests

A tuberculin skin test (TST; PPD-RT23, 2 TU, Statens Serum Institut Kopenhagen) was performed on children in “daycare center A group 1” at the daycare center, the result was documented after 48 to 72 h. A TST with an induration of ≥ 5 mm was considered positive. Symptomatic children and children < 2 years of age (n = 2) were presented to the Pediatric Infectious Disease Clinic of the Charité – Universitätsmedizin Berlin within a week after TB diagnosis of the index, the other children after the TST result were documented.

Children in daycare centers B, C and “daycare center A group 2” received either a TST or a blood drawing for the determination of *M.tb*-specific interferon-gamma release assay (IGRA; QuantiFERON-TB-Gold Plus [QFT-Plus]), whereby the IGRA was evaluated as positive at an interferon-gamma concentration of > 0.35 IU/ml. Children < 5 years of age or with positive TST or IGRA were referred to the clinic.

At the clinic, a standardized questionnaire and examination form was used to record data on age, clinical symptoms (including fever, cough, night sweats, loss of appetite, and rhinitis within the last four weeks), ethnic origin, travel history, BCG (Bacille Calmette-Guérin) vaccination status, TST result, and physical examination. A blood sample was taken from all children for the determination of IGRA, if not already done, transaminases, CRP, creatinine and blood count. All children in “daycare center A group 1” received a chest X-ray (CXR), including a lateral view, regardless of the result of the primary TST, as did all children with a positive TST and/or IGRA in the other daycare groups.

In children with signs of an acute viral infection (cough, runny nose, fever) when presenting in the Pediatric Infectious Disease clinic, a qualitative test for RNA of influenza A, influenza B virus, RSV and SARS-CoV-2 (Xpert Xpress SARS-CoV-2/Flu/RSV”, Cepheid company) from throat swabs was also carried out.

### Prophylaxis and therapy

Children < 5 years of age with negative TST and/or IGRA and an unremarkable CXR—and no prolonged clinical symptoms highly suggestive for TB—were recommended isoniazid prophylaxis (10 mg/kg daily) in accordance with current German guidelines. Prophylaxis was recommended until an IGRA was carried out and showed a negative result at least eight weeks after the last contact with the index [13]. Children who tested positive (IGRA or TST) with an unremarkable CXR and no clinical signs for TB were diagnosed with tuberculosis infection (TBI, formerly known as latent tuberculosis infection; LTBI) and prescribed three months of preventive treatment with isoniazid (10 mg/kg daily) and rifampicin (15 mg/kg daily) [12, 13]. All children with preventive treatment were followed up clinically after two and six weeks. Before preventive treatment was stopped, a CXR was performed to exclude development of TB disease.

Children with suspected pulmonary TB (positive TST and/or IGRA, clinical symptoms suspicious for TB and/or suspicious CXR) underwent microbiological diagnostics (*M.tb* PCR and culture of three fasting gastric aspirates and stool samples) to assess for potential contagiousness. A TB-Flow assay was ordered in children with positive IGRA to differentiate between TBI and TB in the case of an inconclusive TB diagnosis. This assay uses a multivariable score based on the flow cytometric measurement of CD4 T-cell activation and proliferation markers after stimulation with *M.tb*-specific antigens. A score of ≥ 6 indicates TB disease in adult patients: A reliable distinction could be made between TBI (no clinical or radiologic signs of TB in QFT-positive patients) and culture-proven pulmonary or extrapulmonary TB disease [14]. The assay is currently evaluated in children aged 5–17 years by our group (personal communication; Meyer *et al*., manuscript in preparation). We decided to use the TB-Flow assay in the present study to further support pulmonary TB diagnosis in the context of influenza infections in symptomatic children with positive IGRA and/or TST and pathologic CXRs although this assay has not been evaluated in young children. Results must therefore be interpreted with great caution. Children who were diagnosed with TB received therapy in line with the German guidelines, which recommend a 3-drug therapy (isoniazid, rifampicin, pyrazinamide) for two months, followed by a 2-drug therapy (isoniazid, rifampicin) for four months for children with uncomplicated drug-susceptible pulmonary TB [13].

In all groups, regular follow-up visits comprised clinical evaluations for symptoms of TB, medication adverse events and adherence to therapy, including inspection for rifampicin-induced orange coloring of urine samples. All medications were given by parents at home. Parents were strictly instructed to contact us promptly when symptoms suggestive for TB disease occurred during or after treatment.

### Ethics

The study was approved by the Ethics Committee of the Charité—Universitätsmedizin Berlin (No. EA2/066/25).

## Results

62 children were examined, of which 43 children attended daycare center A and 19 children attended daycare centers B and C (Fig. 1).

### Daycare center A group 1

**Fig. 2:**
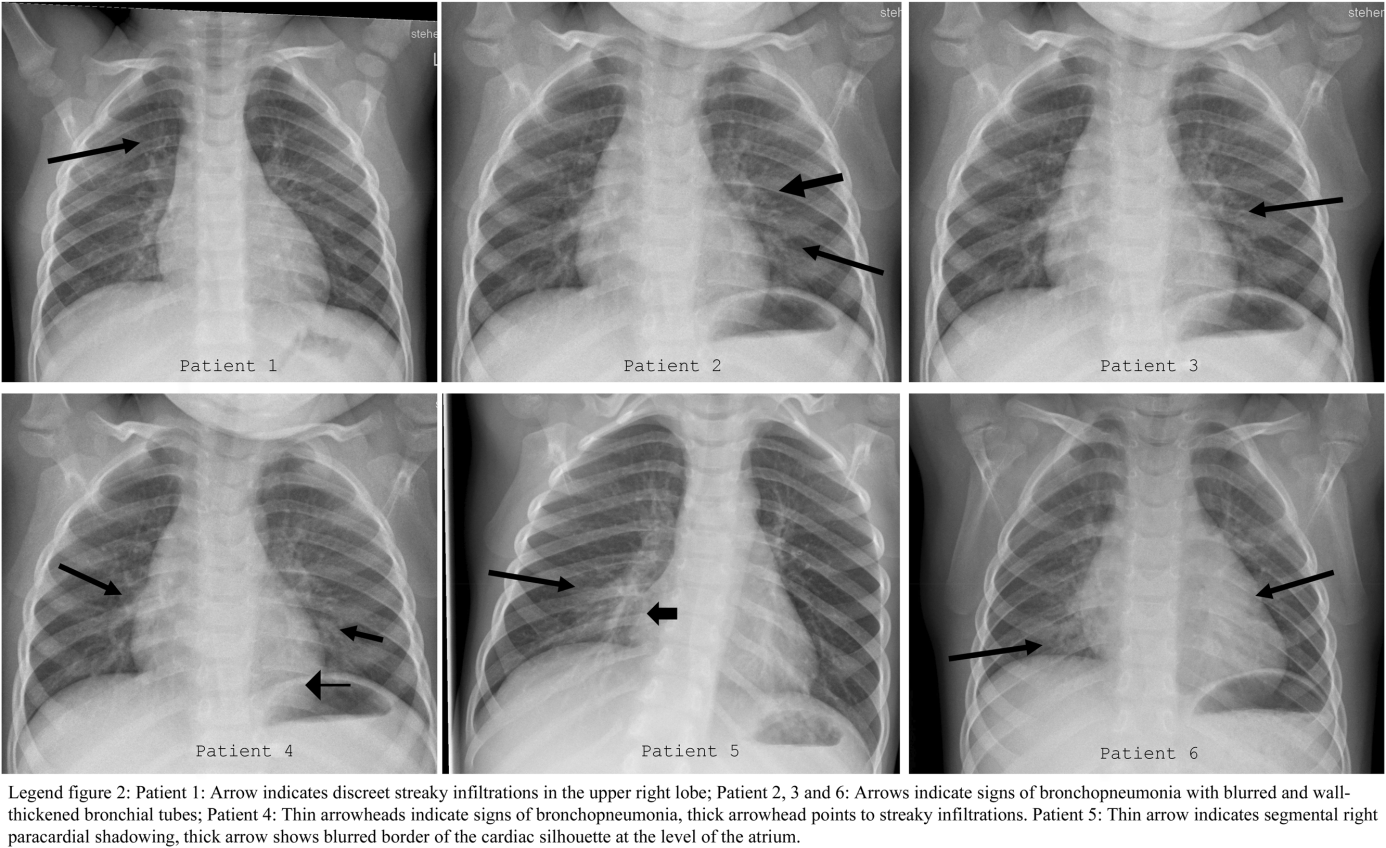
Chest X-rays of six children with pulmonary tuberculosis prior to therapy

The group with the most intensive contact with the index patient consisted of 30 children with a median (range) age of 3.9 (1.7–6.3) years. The initial examination in the daycare center took place at a median (range) of 10 (5–16) days and the initial clinic presentation at a median (range) of 16 (9–17) days after the index patient’s TB diagnosis. Of the 30 children, 28 (93.3%) were born in Germany, two in Ukraine, no child was BCG-vaccinated, all children had a normal nutritional status, the travel history for potential TB exposure was unremarkable in 23/30 (76.7%) children. Fever and cough within the last four weeks were reported in 27/30 (90%) of the children. In nine of the 30 tested children TST and/or IGRA were positive at baseline, there were no discordant results, and additional two children had an indeterminate result of the QFT-Plus test (Table 1). Four of these children were diagnosed with TBI at first presentation and five with pulmonary TB. In another child, two years of age (P3), pulmonary TB was diagnosed one month after the end of INH prophylaxis, following a single negative TST test and two negative IGRA tests (see Fig. 2 for radiology images and footnote for Table 2 for other clinical findings). A TB-Flow assay [14] was performed as an additional tool to confirm the clinical diagnosis of TB—although not evaluated for this age group—in four children with suspected pulmonary TB, two children had a score of 11, two children had a score of 6 and 7, respectively.

**Table 1:** Results of the initial and follow-up tuberculosis screening (children in daycare center A group 1)

Age group	n	TST positive (Induration ≥ 5 mm)	QFT-Plus positive	TST and/or IGRA positive	Cough and/or fever within 4 weeks	Chest X-ray normal
< 5 years	22	6/20 (30%)	3/2 (14.3%)*	6/22 (27.3%)	22 (100%)	14/22 (63.6%)
≥ 5 years	8	3/8 (37.5%)	2/8 (25%)	3/8 (37.5%)	5 (62.5%)	7/8 (87.5%)
Total	30	9/28 (32.1%)	5/29 (17.2%)	9/30 (30%)	27 (90%)	21/30 (70%)

*TST* Tuberculin skin test, *QFT-Plus* QuantiFERON-TB gold plus

^*^QFT-Plus indeterminate in two other children due to high basal interferon-gamma production, both children had a score of 11 in the TB-Flow assay

**Table 2:** Characteristics of children with tuberculosis disease and tuberculosis infection (daycare center A group 1)

Child	Age	TST-result	TST (mm)	QFT-Plus result	TB1 IE/ml	TB2 IE/ml	TB-Flow score	Clinical symptoms	Chest X-ray findings	Virus diagnostics	Diagnosis
P1	2.3	Positive	10	Positive	> 10.00	8.12	n. d	Cough for 6 weeks	Infiltrates	n. d	TB
P2	2.6	Positive	10	n. a	–	–	11	Cough > 3 weeks	Bronchopneumonia	Influenza A	TB
P3	2.6	Negative	0	Negative *Negative	0.00 0.13	0.01 0.24	n. d *n. d	Cough > 3 weeks, fever 3 days	Peribronchitis	Influenza A	TB*
P4	2.9	Positive	18	n. a	–	–	11	Cough > 4 weeks	Peribronchitis	Negative	TB
P5	3.2	Positive	13	n. d	–	–	7	Cough	Hilar lymph-adenopathy	Negative	TB
P6	3.3	Positive	14	Positive	> 10.00	6.79	6	Fever > 5 days, cough	Bronchopneumonia	Influenza A	TB
P7	5.3	Positive	12	n. d	–	–	n. d	Cough	Peribronchitis	n. d	TBI
P8	5.6	Positive	8	Positive	5.67	5.77	n. d	Cough	Normal	n. d	TBI
P9	5.6	Positive	13	Positive	7.77	9.6	n. d	None	Normal	n. d	TBI

*TST* Tuberculin skin test, *QFT-Plus* QuantiFERON-TB Gold Plus, *n. d*. Not done; *n. a*. Not analyzable with high basal interferon-gamma synthesis in negative control

^*^Patient P3: QFT-Plus control negative eight weeks after TB exposure before completion of isoniazid prophylaxis, 12 weeks after TB exposure development of productive cough, fever up to 41 °C, QFT indeterminate, TB-ELISPOT positive, TB-Flow Assay Score: 8, chest X-ray: signs of peribronchitis, three months after the start of therapy, cough symptoms again, radiological atelectasis formation in the right upper lobe, MRI: mediastinal lymphadenopathy and lymph node perforation, bronchoscopic molecular genetic detection of *M.tuberculosis*—complex DNA, genotypically RMP-sensitive, microscopy and culture negative

Isoniazid prophylaxis was recommended in 16 children < 5 years of age with initially negative TST and/or IGRA (see Table 1). Three of these children, all over two years of age, received no prophylaxis due to parental concerns. Four children with TBI received preventive treatment with isoniazid and rifampicin, one of these children developed an isoniazid induced skin rash and subsequently received only rifampicin for 4 months.

All children (n = 21) with initially negative TST and/or IGRA had a negative IGRA at follow-up 59–73 days after the last contact to the index.

### Daycare center A group 2

13 children with a median (range) age of 5.2 (2.3–7.5) years were tested once using TST or IGRA at a median (range) of 31 (26–33) days after the index patient was diagnosed with TB or five months after the last contact. Only one 7 year-old boy without risk factors for TB, had a positive IGRA, TBI was diagnosed and a nine month preventive therapy with isoniazid was carried out due to rifampicin intolerance.

### Daycare center B and C

19 children with a median (range) age of 4 (2–6) years were tested once by TST 11–13 weeks after TB exposure. All children had a negative test result.

### Children diagnosed with TB

All six children (median [range] age 2.7 [2.3–3.3] years) had pulmonary TB and attended “daycare center A group 1”. Neither fasting gastric aspirates nor stool samples showed molecular genetic or cultural evidence of *M.tb*. The results of TST, IGRA, TB-Flow assay, and chest X-rays are shown in Table 2. A nasopharyngeal swab was performed in five children with suspected TB and symptoms of an acute viral infection; in three cases, influenza A virus was detected.

### Therapy

The five children who were diagnosed with TB on initial examination received a 3-drug therapy (isoniazid, rifampicin, pyrazinamide) for two months, followed by a 2-drug therapy (isoniazid, rifampicin) for a further 4–7 months [13]. A shortened duration of therapy according to the WHO guideline 09/2022 [15] was considered in two children, but due to persistent radiological abnormalities after four months of treatment, the 2-drug therapy was continued for a further two months. One child (P3; clinical course: see footnote of Table 2), in whom TB was not diagnosed until five weeks after the end of chemoprophylaxis, initially received a 4-drug therapy with isoniazid, rifampicin, pyrazinamide, and ethambutol for two months, followed by a 2-drug therapy (isoniazid, rifampicin) for additional seven months due to a complicated course with mediastinal lymph node perforation and atelectasis formation. Ethambutol was added to the initial regimen due to concerns for development of drug resistance with prolonged monotherapy with isoniazid.

**Fig. 3:**
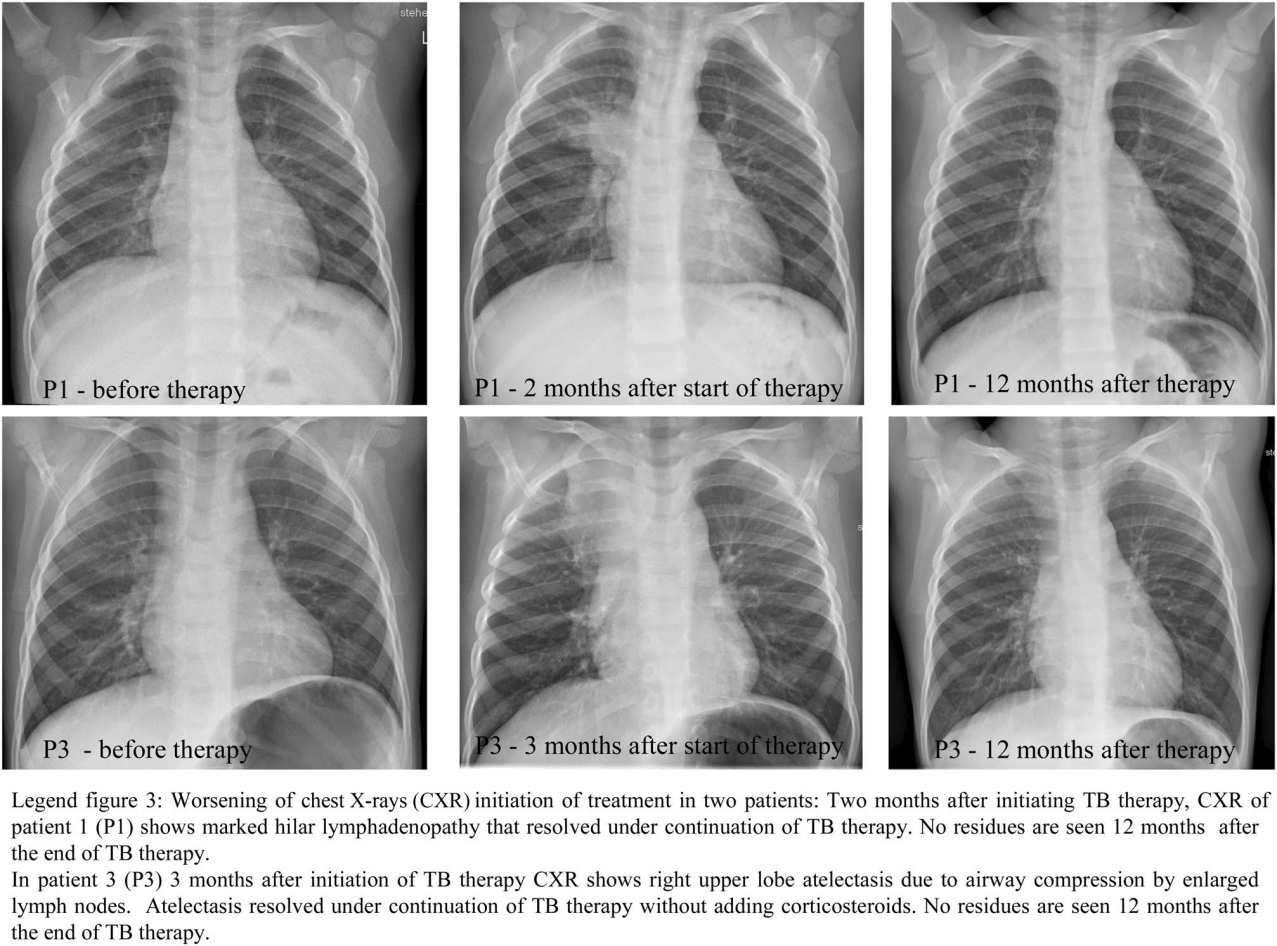
Worsening of chest X-rays after initiation of treatment (paradoxical reaction)

Two children (P1 and P3) showed a worsening of the findings radiologically two and three months after the start of treatment in the sense of a paradoxical reaction (Fig. 3).

### Outcome

After a treatment period of 6–9 months, all six children were clinically asymptomatic, in three of the six children mild residual radiological changes were detectable at the end of treatment, a check-up after a further 12 months showed no abnormalities in either the children with pulmonary TB or those with TBI.

## Discussion

After prolonged TB exposure, 10/30 (33.3%) children with the most intensive contact had positive TST and/or IGRA results, indicating *M.tb* infection. Six of these children were diagnosed with pulmonary TB based on additional pathological CXR findings. As there was no evidence of other sources of infection, it can be assumed that all children were infected by the sick employee. In contrast, no children in groups with brief contact to the index patient were infected or ill, and only one of 13 children from the same daycare center, where last contact had been six months before the index patient was diagnosed with TB, showed evidence of infection.

Comparable infection rates have been described in other TB outbreaks in daycare centers. In Sweden, 60% of infants became infected after prolonged exposure to TB by a staff member, with 28.3% ultimately diagnosed with TB [10]. In Italy, 28.6% (29/103) of young children were infected and 12.6% developed TB after delayed diagnosis of TB in a staff member [9]. An outbreak in San Francisco, USA, affected 32% (9/28) of children, with the majority being young children (median age 3.6 years) [8]. A Norwegian study demonstrated that TB exposed children with brief contacts to an index in a daycare setting had a low risk of infection [16]. Data on the frequencies and extend of TB outbreaks in schools or day care centers in TB endemic countries have not been published recently.

TB in young children can be effectively prevented through prompt examination and prophylactic or preventive treatment after TB exposure [5, 6, 17]. However, this is only possible if the index case is diagnosed early. In our case and other studies, the delayed diagnosis of TB in the index patient favored an outbreak [8–10]. Previous studies by our group showed that delayed TB diagnoses in symptomatic adults represents a “missed opportunity” for preventing TB in children [18]. Measures to prevent TB outbreaks in daycare centers could include routine pre-employment TB screening of childhood educators irrespective of TB risk factors. In addition, existing recommendations for the further elimination of TB in countries with low TB incidence should be implemented more consistently [19].

Germany is a low-incidence country for TB and affected patients usually have a history of travel to or migration from TB endemic countries or immunological risk factors. Our index patient had no defined risk factors, therefore, TB was not considered a differential diagnosis for the progressive, chronic cough. During the SARS-CoV-2 pandemic, similar misinterpretation of chronic cough contributed to delayed TB diagnoses, leading to prolonged exposure of contact persons [20].

The clinical symptoms of TB in young children are usually nonspecific. Particularly in the winter months, when viral respiratory infections are common, clinical and radiological diagnosis is challenging. The diagnosis can often only be made or suspected based on clinical and radiological findings in combination with results of immune-based TB tests (IGRA or TST). Pathogen detection to confirm the diagnosis and assess contagiousness should always be attempted, although this is often not successful in young children [21]. As no single clinical, microbiological or radiological parameter can rule out TB, treatment according to guidelines is indicated in cases of doubt after significant exposure and suspected TB. The high rate of circulating respiratory viruses in the group complicated the diagnosis of pulmonary TB, since neither clinical symptoms nor radiologic findings can clearly differentiate between a respiratory viral infection and pulmonary tuberculosis. In the absence of microbiologic or PCR *M.tb* detection (except in one patient) affected children have to be classified as ‘likely or unconfirmed TB disease’.

Paradoxical reactions with worsening of radiologic findings following initiation of TB therapy were seen in two of our patients (see Fig. 3). Paradoxical reactions (synonymous for post-treatment inflammatory responses) are defined as worsening of clinical, radiologic or laboratory parameters in patients receiving effective TB therapy and are reported in up to 10% of non-HIV-infected children[22–25]. Young age, male gender and non-BCG vaccination status are described as risk factors [24, 25]. Corticosteroids may be of therapeutical use (for review see [23]) but are not generally recommended for pulmonary TB by WHO or national guidelines [13]. In our study group, both children with paradoxical reaction improved without adding corticosteroid therapy.

Even after two negative tests following TB exposure, new radiological and immunological evaluations should be conducted if nonspecific signs, such as inappetence or prolonged fever appear, as demonstrated by the case of patient P3. This raises the question of whether an IGRA re-test eight weeks after last TB exposure is a sufficient interval to rule out TBI. As recommended by Kyaw* et al*., a 10 weeks interval might be more sensitive in detecting TBI and should be considered in future national guidelines [26].

In our study group, 90% of the children suffered from a respiratory tract infection on initial presentation and/or within the preceding four weeks. In three out of five symptomatic children with TB, we were able to detect influenza A by molecular genetic testing in throat swabs. This suggests that many children may have had a concurrent influenza infection during TB exposure or after infection with *M.tb*. Epidemiological data and murine studies show that co-infection with influenza increases morbidity, mycobacterial pathogen load and mortality [27–30]. Historical observations, dating back to the Spanish flu, further support this finding [31]. The increased risk of an *M.tb* infection as well as a more rapid progression after infection by an influenza co-infection may therefore have contributed to the high infection and disease rate in the outbreak described.

Data from studies in adults highlight the risk of long-term sequalae associated with treated pulmonary TB, collectively referred to as “Post-Tuberculosis Lung Disease”, which is associated with increased mortality [32]. While childhood TB typically has a favorable outcome when treated early, studies also indicate a relevant risk of long-term sequalae [33, 34]. A meta-analysis revealed that around 19% of children and adolescents had radiological sequalae after treatment for pulmonary TB [33]. In our study group, all six children with TB were clinically asymptomatic at the end of treatment, radiological residuals were detected in two of the six children at the end of treatment, and after a further 12 months all children had normal radiological findings. The early diagnosis of TB as part of the contact investigation and immediate initiation of treatment may have prevented long term sequalae.

## Conclusion

This TB outbreak demonstrates the importance of early diagnosis of TB in symptomatic adult patients and rapid contact investigation for TB prevention and treatment, particularly in young children at risk. Close cooperation between public health departments and specialized infectious disease centers is essential in outbreak situations. Delayed TB diagnosis in adults is an avoidable risk factor for childhood TB in low-incidence countries (4). Prolonged coughing should therefore trigger a prompt investigation for TB, regardless of occupation, age or ethnicity.

## Data Availability

No datasets were generated or analysed during the current study.
